# Removal of Strong Odors from the Hands

**Published:** 1878-02

**Authors:** 


### REMOVAL OF STRONG ODORS FROM THE HANDS.

The *Schweizerische Wochenschrift fur Pharmacie*, has a communication from F. Schneider, in which he states that ground mustard, mixed with a little water, is an excellent agent for cleansing the hands after handling ordorous substances, such as cod liver oil, musk, valerianic acid, and its salts. Scale pans and vessels may also be readily freed from odor by the same method.

A. Huber states that all oily seeds, when powdered, answer this purpose. The explanation of this action is somewhat doubtful, but it is not improbable that the ordorous bodies are dissolved by the fatty oil of the seed, and emulsionized by the contact with water. In the case of bitter almonds and mustard, the development of ethereal oil, under the influence of water, may perhaps be an additional help to destroy foreign odors. The author mentions that the smell of carbolic acid may be removed by rubbing the hands with flaxseed meal, and that cod liver oil bottles may be cleansed with a little of the same, or olive oil.

